# Glide path enlargement of curved molar canals using HyFlex EDM glide path file versus PathFile: a comparative study of preparation time and postoperative pain

**DOI:** 10.1186/s12903-021-01512-4

**Published:** 2021-03-23

**Authors:** Yi Han, Xiao-Mei Hou

**Affiliations:** The Second Dental Center of Peking University School of Stomatology, No. 66 Anli Road, Chaoyang District, Beijing, 100101 China

**Keywords:** Glide path, Postoperative pain, HyFlex EDM glide path file

## Abstract

**Background:**

This randomized clinical trial aimed to compare the preparation time and severity of postoperative pain between HyFlex Electric Discharge Machine (EDM) glide path file (GPF) and PathFile.

**Methods:**

Eighty patients whose molar teeth had at least one severely curved canal were treated by the same specialist. After access cavity preparation, the patients were randomly assigned to receive glide path enlargement with either HyFlex EDM GPF or PathFile. ProTaper Next X1 and X2 files were used to prepare the canals. The time of preparation was assessed and the severity of postoperative pain over the next 7 days was recorded. The preparation time and the postoperative pain scores were compared using the Linear Mixed Models (*P* ≤ 0.05).

**Results:**

Glide path enlargement time was significantly shorter with HyFlex EDM GPF (27.828 ± 2.345 s) than with PathFile (48.942 ± 2.864 s) (*P* < 0.001). The highest postoperative pain score was recorded on the first day and the pain decreased over time in both groups. HyFlex EDM GPF group patients reported significantly less postoperative pain than PathFile group patients overall (*P* < 0.001).

**Conclusions:**

Postoperative pain and glide path preparation time could be reduced by using HyFlex EDM GPF system.

*Trial registration* PKUSSNCT, PKUSSNCT-17B12, Registered 24 October 2017.

## Background

Endodontic postoperative pain is a sensation of discomfort experienced by 25–40% of patients after root canal treatment [1, 2]. Post-endodontic pain usually occurs during the first 2 days after treatment and generally diminishes within a few hours. However, in some cases, the pain may persists for several days. Multiple factors contribute to the sensation and severity of the pain. Clinical investigation of the postoperative pain is challenging [3]. Mechanical, chemical and microbial damage to periapical tissue is considered to be the main cause of acute periapical periodontitis [4]. In the process of root canal preparation, irritants such as infected debris can be squeezed into the periapical tissue to induce acute inflammatory reaction. So far, all the instruments and techniques of root canal preparation are related to a certain degree of fragment extrusion [5, 6]. Glide path preparation, establishment of apical patency and endodontic status are important factors that affect and reduce the incidence of the post-treatment pain [3, 7–9].

The endodontic glide path is the smooth tunnel created from the orifice of the root canal to the physiological terminus of the root apex, which facilitates cleaning and shaping during root canal treatment [10]. Preliminary canal enlargement is also considered an important step because it can reduce torsional stress and further improve the life of the shaping instruments. This also reduces the risk of procedural errors such as ledge formation, canal transportation and perforations [11, 12]. Glide path enlargement can be performed with precurved stainless steel K-files or nickel–titanium (NiTi) glide path files (GPFs). In 2017, Alovisi et al. and Paleker et al. demonstrated that NiTi rotary instruments enable faster and safer preparation of a glide path that respects the original canal anatomy [13, 14].

The HyFlex EDM (Coltene/Whaledent, Altstätten, Switzerland) NiTi GPF is a GPF file system produced using an innovative manufacturing process called Electrical Discharge Machine with a controlled memory (CM) wire. In EDM, instead of conventional grinding, electric discharges are used to shape the file via melting and vaporization of the material [15]. This method creates a cratered surface, which further increases the file’s cyclic fatigue resistance and lifetime [16]. The HyFlex EDM GPF consists of a single file with a tip size of 10 and a 5% taper. The cross-section of the HyFlex EDM GPF varies along its length, being quadratic at the tip, trapezoidal in the middle, and triangular at the shaft. The flexibility of the HyFlex EDM GPF confers this instrument the ability to maintain the apical canal curvature despite its greater taper [17].

The PathFile (Dentsply Sirona, Ballaigues, Switzerland) NiTi rotary system consists of three file systems, which are made of conventional NiTi alloy with different tip sizes such as 13#, 16#, and 19#. The files have square cross-sections, with four cutting edges and a 2% taper. Glide path preparation with PathFile promotes less deviation from the original canal anatomy when compared with hand-operated instruments [18].

The effect of GPFs on postoperative pain has not been adequately investigated. Therefore, the aim of this study was to compare the preparation time and severity of postoperative pain with two glide path preparation systems—HyFlex EDM GPF versus PathFile.

## Methods

The study was approved by the local university clinical research ethics committee with vide letter no. PKUSSIRB-201735063. Prior to this clinical trial, we did an in vitro simulated root canal study. We compared the shaping ability and preparation time of HyFlex EDM GPF with PathFile in simulated root canals. Using PASS software, the required sample size in this study was determined by comparing the mean preparation time for HyFlex EDM GPF and PathFile. In this study, two-tailed hypothesis test was used, with a test level of 0.05 and a power of 90%.

According to existing literatures on evaluation preparation time, the mean of HyFlex EDM GPF in the experimental group was 196.825 s, with standard deviation of 10.328 s, and the mean of the control group was 229.633 s, with standard deviation of 16.870 s. The sample size of each group was calculated according to 1:1 matching. Further stratified comparison of samples was considered in the analysis, and the sample size should be at least 8–10 times the number of variables in the multi-factor analysis. Therefore, appropriately increasing the sample size was considered, and the sample size of 40 people in each group designed in this study can basically meet the needs of analysis.

The sample consisted of 80 patients who were treated at the Second Dental Center of Peking University School of Stomatology between June 2018 and February 2019. The following inclusion criteria had to be fulfilled by the patients to be enrolled in the study: (1) More than 18 years old. (2) No previous history of systemic diseases. (3) Selected for root canal treatment for asymptomatic or symptomatic irreversible pulpitis in the maxillary or mandibular first or second molars with at least one severely curved canal (> 25°, measured by ImageJ2x). The diagnosis of symptomatic irreversible pulpitis was based on positive pulp sensibility test results of a cold test and an electric pulp test, presence of spontaneous pain, and deep caries or extensive restorations exposing the pulp. The diagnosis of asymptomatic irreversible pulpitis was based on no clinical symptoms and presence of a deep caries that will result in a pulp exposure following removal. The diagnosis was recorded after access cavity preparation with the presence of bleeding. Periapical radiographs were taken for each patient using a digital radiologic system (Soredex, Finland). Curvatures of the curved canals were analysed by 2 blinded examiners who were specialists in endodontics. If conflicts existed, a third opinion was obtained from another endodontist. Kappa test using a case series of interexaminer reliability above 0.90, was reached prior to the trial.

The exclusion criteria were (1) Pulp necrosis, with or without apical periodontitis, based on negative pulp cold and electirc tests and periradicular radiographic feature, diagnosis recorded after access cavity preparation without bleeding. (2) Acute or chronic apical abscess with signs of systemic infection. (3) Allergic to local anesthetic agents. (4) Progressive periodontal disease at any stage (clinical attachment loss ≥ 5 mm, probing depth ≥ 6 mm, or furcation involvement class II or III). (5) Taking analgesic, antibiotic or anti-inflammatory drugs during the 7 days prior to the treatment.

Before the procedure, pulpal and periradicular status was evaluated by means of a cold test and an electric pulp tester (Diagnostic, SybronEndo, USA), reactions to palpation and percussion also recorded. Periodontal probing was performed and periradicular radiographic image was taken and analysed. These datas were recorded on each patient’s clinical note, together with baselined emographic information (Table 1). At this stage, a sealed envelope containing the randomization code was opened and the patient was assigned to receive glide path enlargement with either the HyFlex EDM GPF or PathFile, with 40 patients in each group. Patients were blinded regarding the file system utilized.

**Table 1:** Characteristics of the two groups

Demographic data	HyFlex EDM GPF group	PathFile group	*P* value*
Age, years	48.1 ± 14.1	45.5 ± 14.4	*P* > 0.05
Male	12	11	
Female	28	29	
First molar	19	19	
Second molar	21	21	
Angle of root canal	28.8° ± 6.8°	26.5° ± 8.3°	
Asymptomatic irreversible pulpitis	9	7	
Symptomatic irreversible pulpitis	31	33	

Data are mean ± standard deviation or number

*P > 0.05, statistically not significant

## HyFlex EDM GPF group

The working length (WL) was determined using the Root ZX apex locator (SM-DP-ZX, Morita, China) by inserting a size 10 K-file into the root canal up to the apical foramen. The WL was confirmed radio-graphically and was also repeatedly checked during the treatment procedure. The HyFlex EDM GPF (size 10/0.05) was operated with an endomotor (X-SMART, Dentsply Maillefer, Switzerland) in continuous rotation at a speed of 300 rpm and 1.8 N·cm of torque, according to the manufacturer’s instructions. Irrigation with 5.25% NaOCl and 17% EDTA was carried out during the glide path enlargement and the preparation time was recorded from the insertion of the GPF till the completion of the enlargement, including irrigation time during the enlargement. After the instrumentation was completed, the root canal was irrigated with 5 mL of 5.25% NaOCl and 5 mL of 17% EDTA.

## PathFile group

The WL was determined as described above in the HyFlex EDM GPF group. The PathFile (size 13/0.02 and 16/0.02) instruments were operated using the same endomotor (X-SMART) at a speed of 300 rpm and 5 N·cm of torque. Irrigation with 5.25% NaOCl and 17% EDTA was carried out during the glide path enlargement and the preparation time was recorded from the insertion of the PathFile 13/0.02 till the completion of the PathFile 16/0.02 enlargement, including the irrigation time during the enlargement and the time required to change files. After enlargement, the root canal was irrigated with 5 mL of 5.25% NaOCl and 5 mL of 17% EDTA.

After glide path creation, subsequent endodontic procedures were standardized as per the protocol. The root canals were then prepared with X1 and X2 instruments by using the ProTaper Next (Dentsply Maillefer, Switzerland) rotary instrumentation system and then irrigated with 5.25% NaOCl and 17% EDTA.

The preparation time of the severely curved canal for the GPFs and ProTaper Next was recorded. Only the time used for active instrumentation such as checking the WL, cleaning the flutes of the instruments, and irrigation were included. The time taken to change files was not considered. The preparation time was recorded by a nurse and other canals for the same molar were prepared using only the ProTaper Next.

Accordingly, the prepared root canals were irrigated again with 10 mL of 5.25% NaOCl with a 30-gauge needle syringe and then dried with sterile paper points. Calcium hydroxide (Ca(OH)_2_) was placed in the canals, temporary filling was performed, and an appointment was scheduled to complete the subsequent root canal procedure. Finally, all patients were informed about the possible pain and were instructed how to complete a visual analog scale (VAS) to determine their postoperative pain scores. The pain score was recorded on a scale of 0–10. Pain intensity was categorized as no pain (0), mild pain (1–3), moderate pain (4–6), and severe pain (7–10). The patients were instructed to record postoperative pain intensity twice daily (morning and evening) for 1 week using a visual analog scale (VAS) on paper. They were recommended to take ibuprofen 600 mg when required. All patients returned the original copies of the VAS form on the next visit.

### Statistical analysis

SPSS version 20.0 (IBM Corp, Armonk, NY, USA) was used for statistical analysis. The distributions of age, sex, the tooth treated, and the angle of root canal curvature in the two treatment groups were compared by using the chi-square test or t test. The preparation time and the postoperative pain scores were compared using the Linear Mixed Models. Statistical significance was recognized at *P* ≤ 0.05 (two-tailed). Considering that multiple pain measurements were performed for the same individual within 7 days after the preparation of the curved root canal, the Linear Mixed Models was used to analyze the differences between groups and the trend of pain changes over time in different groups.

## Results

The age of the patients was between 23 and 73 years old, 48.1 ± 14.1 (12 men and 28 women) in the HyFlex EDM GPF group compared to 45.5 ± 14.4 (11 men and 29 women) in the PathFile group. Regarding the tooth type, 19 first molars and 21 second molars were included in each group. The angle of the root canal was 28.8° ± 6.8° in the HyFlex EDM GPF group and 26.5° ± 8.3° in the PathFile group. Demographic variables such as age and sex and clinical variables such as the tooth type and the angle of the root canal curvature were similarly distributed in the two treatment groups (all *P* > 0.05, Table 1).

Table 2 presented the mean total time for glide path preparation using both methods. Glide path enlargement time was significantly shorter with HyFlex EDM GPF (27.828 ± 2.345 s) than with PathFile (48.942 ± 2.864 s) (*P* < 0.001).

**Table 2:** Preparation times (in seconds) in the two groups

Group	n	Time for glide path preparation	Time for ProTaper Next preparation	Total time
HyFlex EDM GPF group	40	27.828 ± 2.345	69.432 ± 3.964	97.260 ± 6.015
PathFile group	40	48.942 ± 2.864	73.889 ± 4.476	122.830 ± 6.493
*F statistic*		1559.656	6.752	333.802
*P*		< 0.001	0.011	< 0.001

Values are means ± standard deviations

No patients reported endodontic interappointment emergencies and analgesics intake. There was no severe pain in both groups during the first week after the procedure. The highest postoperative pain score was recorded on the first day. All of the patients had mild or moderate pain on the first day, but the pain decreased over time in both groups (Table 3).

**Table 3:** Descriptive statistics of postoperative pain results for HyFlex EDM GPF and PathFile groups

VAS score	Treatment group	Postoperative follow-up interval													
		AM1	PM1	AM2	PM2	AM3	PM3	AM4	PM4	AM5	PM5	AM6	PM6	AM7	PM7
No pain (0)	HyFlex EDM GPF	0	1	1	1	1	1	5	6	10	11	16	16	21	21
	PathFile	0	0	0	0	1	1	1	1	3	3	7	9	14	14
Mild pain (1–3)	HyFlex EDM GPF	18	19	28	29	31	34	32	31	28	27	23	23	18	18
	PathFile	13	13	21	23	26	27	28	28	29	29	29	28	25	25
Moderate pain (4–6)	HyFlex EDM GPF	22	20	11	10	8	5	3	3	2	2	1	1	1	1
	PathFile	27	27	19	17	13	12	11	11	8	8	4	3	1	1
Severe pain (7–10)	HyFlex EDM GPF	0	0	0	0	0	0	0	0	0	0	0	0	0	0
	PathFile	0	0	0	0	0	0	0	0	0	0	0	0	0	0

**Fig. 1:**
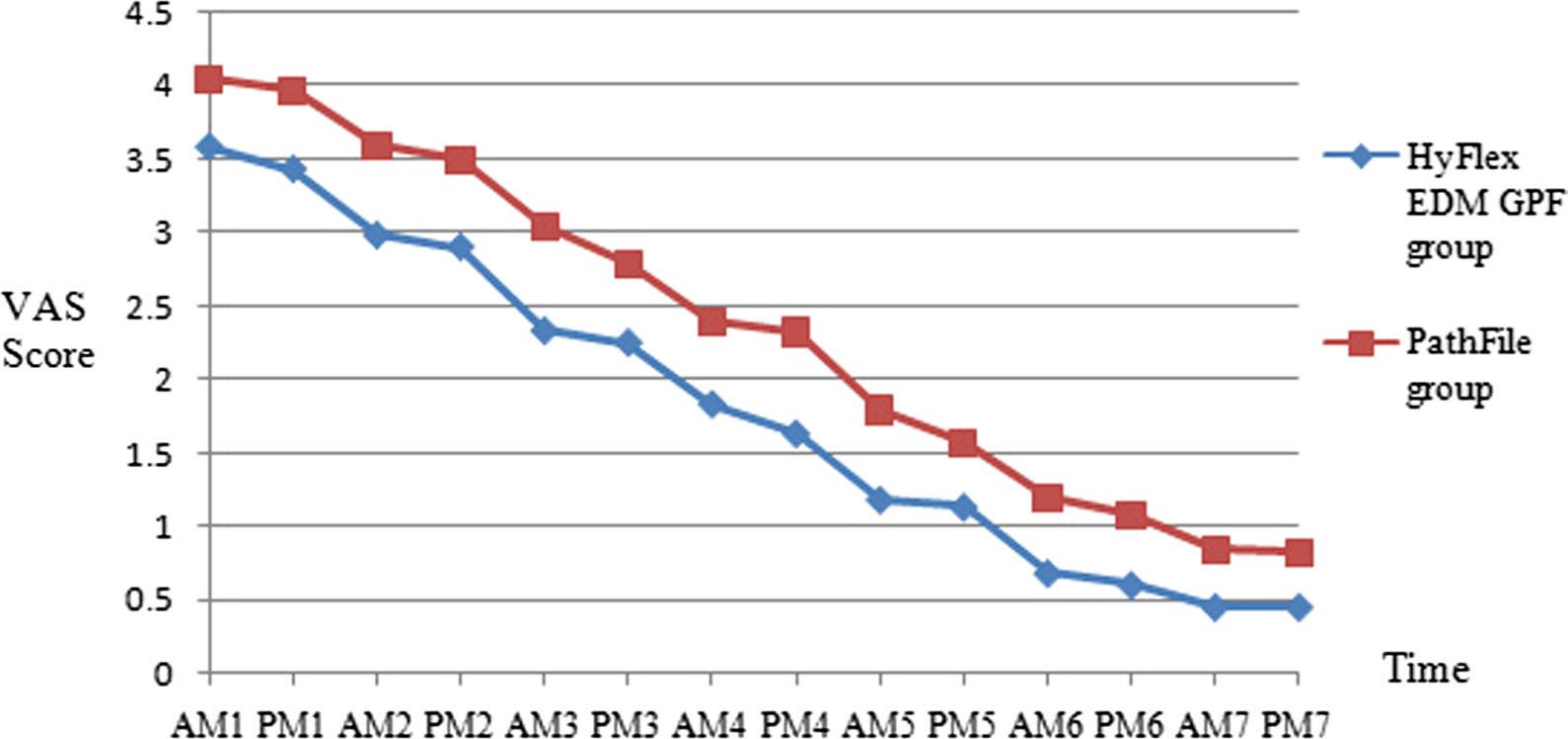
Postoperative VAS scores in the two groups at different time points

The horizontal axis for VAS was the recorded time, which was recorded twice daily (morning and evening) for 1 week after root canal preparation. The vertical axis consisted of the mean VAS scores. HyFlex EDM GPF group patients reported significantly less postoperative pain than PathFile group patients overall, with a significant *P* value of less than 0.05 (Fig. 1).

The results of the Linear Mixed Models showed that there were statistically significant differences in pain scores between the HyFlex EDM GPF group and the PathFile group (*F* = 91.223, *P* < 0.001). There were statistically significant differences in pain scores at 14 different time after root canal instrumentation (*F* = 1442.987, *P* < 0.001). The HyFlex EDM GPF group had an average pain score 0.54 lower than the PathFile group (*F* = −9.551, *P* < 0.001).

## Discussion

This randomized clinical trial aimed to compare the HyFlex EDM GPF versus PathFile with regard to the glide path preparation time and postoperative pain. The mean preparation time was significantly shorter with HyFlex EDM GPF than with PathFile. This shorter time can be explained by the fact that two instruments are used in the PathFile system, whereas only a single instrument is used in the HyFlex EDM GPF system. A recent study by Kirchhoff et al. compared glide path preparation using ProGlider and PathFile in curved mandibular canals. The ProGlider is a single-instrument system, whereas the PathFile is a multi-file system. The authors reported that faster glide path preparation was achieved with the single instrument system [12]. Another study also compared the glide path preparation time with the ProGlider file, PathFile, X-Plorer Canal Navigation NiTi file, and stainless-steel K-file [19]. They also reported that there was a significantly shorter preparation time with the ProGlider file compared to the other systems.

Interestingly, the present study found that the time for ProTaper Next preparation was also significantly shorter in the HyFlex EDM GPF group than in the PathFile group as the preliminary enlargement of the root canal at the coronal and middle portions reduced torsional stress during subsequent root canal preparation, and therefore the WL could be achieved more easily with the ProTaper Next. It should also be noted that the larger taper (up to 5%) of the HyFlex EDM GPF creates a larger canal at the coronal and middle portions.

Postoperative pain is considered to have an important impact on the quality of life, so the proper management of pain is very important. Although postoperative pain is multifactorial, root canal preparation technique is considered to play a major role in the pain [9, 20, 21]. During chemomechanical preparation, infected debris and extruded bacteria may be transported to periapical tissue, where they can induce acute inflammation in periapical area [22]. In this study, the WL and the type and amount of irrigating solution were controlled as these factors can affect debris extrusion, which may influence the reaction of periodontal ligament. Therefore, in all cases, efforts are made to reduce overpreparation by using Root ZX apex locator and radiographic control WL. The side ventilation irrigation needle is used to transport the irrigating agent to the top safely and effectively, which limits the extrusion of the irrigating solution. The present study showed that the significant differences in postoperative pain scores between GPFs and ProTaper Next might be due to the extrusion of debris.

In the present study, the highest postoperative pain score was recorded on the first day. All patients had mild or moderate pain on the first day and the pain decreased over time in both groups. Postoperative pain can occur within a few hours or a few days after endodontic treatment [23]. In systematic reviews, the incidence of postoperative pain was reported to range from 25 to 40% [1, 2] and varied between reports according to the type of study (prospective or retrospective), patient selection, diagnosis of pulp and apical periodontitis, and the experience and qualification of the dentist. Only asymptomatic or symptomatic irreversible pulpitis were included in this study. There was no periapical inflammation. The numbers of asymptomatic and symptomatic irreversible pulpitis were similarly distributed in the two treatment groups. Although we did not use the VAS to record preoperative pain, we considered that preoperative pain had little effect on postoperative pain.

Glide path preparation minimizes the possibility of extrusion debris and bacteria into the periapical area, which further reduces postoperative pain, regardless of the type of instrument used for root canal shaping [21, 24]. In the present study, postoperative pain was significantly lower in the HyFlex EDM GPF group compared with that in the PathFile group. The larger taper (up to 5%) of the HyFlex EDM GPF is intended to remove cervical interference from the root canal entrance, providing free access for endodontic instruments and irrigants to the periapical region, which further helps in reducing the extrusion of debris. A study by Ha et al. indicated the progressive taper design of ProGlider, the off-center cross-section of One G, and the alternative-pitch design of ScoutRace were intended to increase the efficiency of debris removal and minimize extrusion during glide path preparation. Debris extrusion was reported to be lower with the ProGlider system than with the PathFile and One G systems [24].

Glide path preparation prior to mechanical enlargement is helpful to reduce the operation errors in the follow-up root canal procedures. Apical transportation and foramen widening may lead to the loss of apical stop, which further causes more postoperative discomfort [25]. In this study, glide path preparation using HyFlex EDM GPF with large taper designs could produce enlargement in the coronal and middle third of the root canal, which will allow subsequently used instruments to more consistently reach the apical foramen and also helps in improving the precision of electronic apex locators for determining the WL.

The HyFlex EDM GPF has significantly greater cyclic fatigue resistance and flexibility than PathFile [15]. The cyclic fatigue resistance for HyFlex EDM was 700% greater than that for HyFlex CM files made of the same alloy. The porous cratered surface of the EDM files was maintained with no change to the cutting blades even after being used in curved root canals [15]. Another study compared the cyclic fatigue resistances amongst different file systems in artificial canals created with HyFlex GPF (Coltene-Whaledent), G Files (Micro-Mega, Besançon, France), ProGlider (Dentsply Sirona), PathFile, and ScoutRaCe (FKG Dentaire, La Chaux-de-Fonds, Switzerland) NiTi rotary GPFs. The authors found that the greatest cyclic fatigue resistance was in HyFlex GPF made of CM alloy [26]. The flexibility and cyclic fatigue resistance of HyFlex EDM GPF confers this instrument the ability to maintain the apical canal curvature despite its greater taper, which may lead to low postoperative pain.

The limitation of this study was that the endodontic dentist was not blinded to the grouping information. Additionally, the type of teeth was not standardized in the study and there was no differentiation between maxillary and mandibular molars or between first and second molars.

## Conclusions

Glide path preparation time is shorter and postoperative pain severity is lower with the HyFlex EDM GPF than with PathFile. Glide path preparation with HyFlex EDM GPF is advantageous to the subsequent NiTi instruments and can reduce postoperative pain. The highest postoperative pain score was recorded on the first day.

## Data Availability

The datasets used and/or analyzed during the current study are available from the corresponding author on reasonable request.

## Abbreviations

GPFs: Glide path files
CM: Controlled memory
